# Maternal risk factors for posterior urethral valves

**DOI:** 10.3389/fped.2023.1110117

**Published:** 2023-04-28

**Authors:** Loes F. M. van der Zanden, Sander Groen in ‘t Woud, Iris A. L. M. van Rooij, Josine S. L. T. Quaedackers, Martijn Steffens, Liesbeth L. L. de Wall, Michiel F. Schreuder, Wout F. J. Feitz, Nel Roeleveld

**Affiliations:** ^1^ Department for Health Evidence, Radboud university medical center, Nijmegen, Netherlands; ^2^ Department of Urology, University Medical Center Groningen, Groningen, Netherlands; ^3^ Department of Urology, Isala, Zwolle, Netherlands; ^4^ Division of Pediatric Urology, Department of Urology, Amalia Children's Hospital, Radboud university medical center, Nijmegen, Netherlands; ^5^ Department of Pediatric Nephrology, Amalia Children's Hospital, Radboud university medical center, Nijmegen, Netherlands

**Keywords:** environment, exposure, maternal, posterior urethral valves (PUV), risk factors

## Abstract

**Introduction:**

Posterior urethral valves (PUV) is a congenital disorder causing an obstruction of the lower urinary tract that affects approximately 1 in 4,000 male live births. PUV is considered a multifactorial disorder, meaning that both genetic and environmental factors are involved in its development. We investigated maternal risk factors for PUV.

**Methods:**

We included 407 PUV patients and 814 controls matched on year of birth from the AGORA data- and biobank and three participating hospitals. Information on potential risk factors (family history of congenital anomalies of the kidney and urinary tract (CAKUT), season of conception, gravidity, subfertility, and conception using assisted reproductive techniques (ART), plus maternal age, body mass index, diabetes, hypertension, smoking, and use of alcohol and folic acid) was derived from maternal questionnaires. After multiple imputation, adjusted odds ratios (aORs) were estimated using conditional logistic regression corrected for minimally sufficient sets of confounders determined using directed acyclic graphs.

**Results:**

A positive family history and low maternal age (<25 years) were associated with PUV development [aORs: 3.3 and 1.7 with 95% confidence intervals (95% CI) 1.4–7.7 and 1.0–2.8, respectively], whereas higher maternal age (>35 years) was associated with a lower risk (aOR: 0.7 95% CI: 0.4–1.0). Maternal preexisting hypertension seemed to increase PUV risk (aOR: 2.1 95% CI: 0.9–5.1), while gestational hypertension seemed to decrease this risk (aOR: 0.6 95% CI: 0.3–1.0). Concerning use of ART, the aORs for the different techniques were all above one, but with very wide 95% CIs including one. None of the other factors studied were associated with PUV development.

**Conclusion:**

Our study showed that family history of CAKUT, low maternal age, and potentially preexisting hypertension were associated with PUV development, whereas higher maternal age and gestational hypertension seemed to be associated with a lower risk. Maternal age and hypertension as well as the possible role of ART in the development of PUV require further research.

## 1. Introduction

Posterior urethral valves (PUV) is a congenital anomaly of the lower urinary tract in boys that impairs urinary flow. It is a prevalent cause of end-stage kidney disease in children (1) and affects approximately 1 in 4,000 live male births (2). The precise embryological mechanism that results in PUV is uncertain, but the most common theory is that PUVs develop when the mesonephric duct fuses with the cloaca in the 4th or 5th week after conception (which equals week 6 or 7 of pregnancy) (3, 4). PUV is considered to be a multifactorial disorder, meaning that both genetic and environmental factors are involved in its development (5).

Little research has been performed on possible risk factors associated with PUV specifically, as most research so far focused on congenital anomalies of the kidney and urinary tract (CAKUT) as a whole (6). Several studies that focussed on CAKUT in general but included patients with obstructive uropathies (such as PUV) or urethral malformations, found associations between CAKUT and maternal factors, such as age (7), obesity (6, 8, 9), gravidity (7), use of assisted reproductive techniques (ART) (6), and diabetes (6–8, 10). However, other studies were unable to confirm the association with obesity (11). Maternal subfertility (6), hypertension (7), smoking (6, 7, 12), and alcohol use (6, 7) did not seem to be associated with CAKUT, while use of folic acid supplements or folic-acid containing multivitamins was found to be protective (6, 13, 14).

Previous studies showed that separate analyses for different CAKUT phenotypes or for higher vs. lower urinary tract anomalies gave dissimilar results (6, 9). For example, high maternal body mass index (BMI) was associated with upper but not lower urinary tract anomalies. Periconceptional folic acid use was associated with some CAKUT phenotypes but not with PUV, while PUV was the only phenotype associated with gestational diabetes (6, 9). More knowledge on the aetiology of PUV is needed, in order to take preventive measures for PUV, such as adaptation in perinatal care. Therefore, we investigated maternal risk factors for PUV in this study.

## 2. Materials and methods

### 2.1. Study participants

AGORA (Aetiologic research into Genetic and Occupational/environmental Risk factors for Anomalies in children) is a large data- and biobank that was initiated in the Radboud university medical center (Radboudumc), Nijmegen, the Netherlands, in which questionnaire data, blood/saliva samples, and phenotypic information are collected from patients with congenital malformations and their parents (15). The AGORA questionnaires are filled out on paper and inquire about demographics, family and pregnancy history, and health and lifestyle in the three months before and during pregnancy.

For the current project, we selected the patients with PUV from the Dutch patient population that we collected for our studies on obstructive uropathy (16). In these studies, we included both patients with ureteropelvic-junction obstruction (UPJO) and PUV who were born in 1981 or later and underwent a pyeloplasty (for UPJO) or valve resection (for PUV) before the age of 18 years from the AGORA data- and biobank. In addition, we searched the medical registry of the Radboudumc to identify more patients born in 1981 or later who underwent a pyeloplasty or valve resection before 18 years of age. We also searched the medical registries of the Isala clinics in Zwolle and the University Medical Center in Groningen (UMCG) where, due to registration issues, we were only able to identify patients treated in 2002 or later. The newly identified patients or their parents (depending on the patients' age) were approached to participate in our obstructive uropathy studies by providing saliva samples and completing the AGORA questionnaires either online or on paper. From all of the participants, only patients who underwent a valve resection were included in the current study.

In 2010–2011, controls were recruited through 39 municipalities, covering the referral areas of the hospitals through which patients were included in AGORA. These villages and cities were asked to provide a random sample of 150 or 300 of their inhabitants in the age range of 0 to 20 years, which was comparable to that of the patient population at time of recruitment. The parents of these children were asked to fill out the same paper questionnaire as the parents of the patients. In 2021, the control population was supplemented with data from children from 28 municipalities born between 2011 and 2021 using a similar approach. In addition, we asked the parents of the patients who completed the questionnaire online to ask parents of a healthy child of similar age to fill out the same online questionnaire. For the current project, we selected only male controls without major structural birth defects as defined by EUROCAT (17).

The Medical Ethics Assessment Committee East Netherlands (METC Oost-Nederland) approved the AGORA data- and biobank (2006-048, 2018-4524 and 2021-13067), while the board of directors of the Isala clinics and the UMCG approved implementation of AGORA in these centers. All participants and/or their parents gave written informed consent for participation in the study.

### 2.2. Risk factors and confounders

Information about potential risk factors, variables to be used in imputation, and possible confounders was derived from the questionnaires. We considered the following potential risk factors for PUV: family history of CAKUT, season of conception, gravidity, subfertility and conception using ART, as well as maternal age, BMI, diabetes, hypertension, smoking, alcohol consumption, and use of folic acid or folic-acid containing multivitamins. Several other factors, such as duration of pregnancy, year of birth, birthweight, ethnicity, and education, were abstracted to be used in imputation or as possible confounding variables.

Family history was defined as self-reported maternal or paternal CAKUT. Season of conception was derived from the date of birth and pregnancy duration. Gravidity was categorized into first or subsequent pregnancy. Couples were considered fertile if the parents indicated in the questionnaire not to be subfertile and the time to pregnancy was less than 12 months. Subfertile couples were divided into a subfertile group that conceived the index pregnancy without ART, a group using intra-uterine insemination (IUI) without using hormones, a group using hormonal treatment with or without IUI, and a group using in-vitro fertilization (IVF) or intracytoplasmic sperm injection (ICSI). Maternal age was categorized into younger (<25 years), average (25–34 years), and older (≥35 years) age and maternal BMI into underweight (<18.5 kg/m^2^), normal weight (18.5–24.9 kg/m^2^), overweight (25.0–29.9 kg/m^2^), and obese (≥ 30.0 kg/m^2^). Diabetes was considered to be preexisting when women reported it from the start of pregnancy and gestational when it was first diagnosed during the index pregnancy. Preexisting hypertension was defined as any hypertension discovered before the 20th week of gestation, in accordance with guidelines from the American College of Obstetricians and Gynecologists (18), while gestational hypertension appeared from week 20 onwards. For maternal smoking and alcohol use, mothers were divided into a group that smoked or used alcohol during the etiologically relevant period (defined as using before pregnancy and not stopping before the sixth week of pregnancy), a group that smoked or used alcohol prior to the etiologically relevant period (defined as any smoking or alcohol use in the three months before or during the first five weeks of pregnancy only), and a group that did not smoke or use alcohol in the three months before and during pregnancy. For maternal use of folic acid supplements or folic acid-containing multivitamins, mothers were divided into a group that used these supplements as recommended (defined as starting usage before pregnancy and not stopping before the eighth week of pregnancy), a group with suboptimal use (defined as using folic acid supplements or folic-acid containing multivitamins in the four weeks before pregnancy or in the first eight weeks of pregnancy, but not the entire period), and a group that did not use these supplements in this period.

### 2.3. Statistical analyses

Each patient was matched to two controls born in the same year to account for differences in year of childbirth between patients and controls. Crude odds ratios (ORs) with 95% confidence intervals (CIs) were estimated for the potential risk factors using conditional logistic regression.

**Figure 1:**
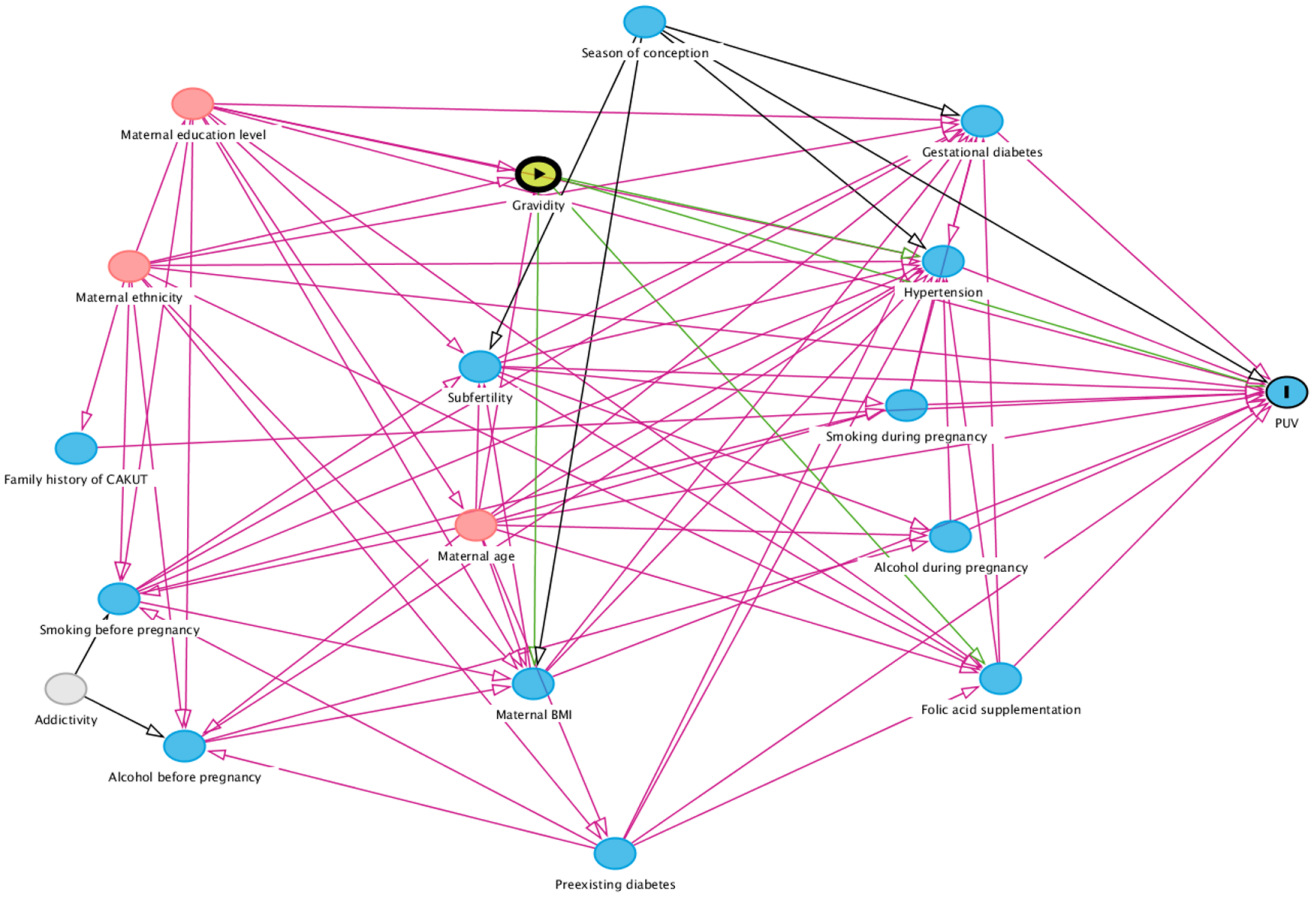
Directed acyclic graph containing all potential risk factors and confounders with their causal relations. Blue circles with black lining: outcome. Blue circles: ancestors of outcome. Pink circles: ancestors of exposure and outcome. Yellow circle: exposure. Grey circle: unobserved variable. BMI, body mass index; CAKUT, congenital anomalies of the kidney and urinary tract; PUV, posterior urethral valves.

For each potential risk factor separately, a minimally sufficient set of confounders was determined using a directed acyclic graph (DAG) created in DAGitty (19). Figure 1 contains the DAG with all our assumptions about causal associations between the potential risk factors and the potential confounders maternal education (divided into low, medium, and high) and ethnicity (defined as European or other). As an example, gravidity was selected as potential risk factor in the DAG in Figure 1, which also shows the minimally sufficient set of confounders for the association between gravidity and PUV.

We used all patients and controls to perform multiple imputation to generate ten imputed datasets. In the imputation, we used all potential risk factors and confounders as well as the outcome, the duration of pregnancy, birthweight, the time between birth and filling out the questionnaire, whether the questionnaire was completed online or on paper, and year of birth. After imputation, adjusted ORs were estimated in the matched patient-control sets using conditional logistic regression corrected for the relevant minimally sufficient set of confounders, plus a variable indicating whether the questionnaire was completed online or on paper. Adjusted ORs were not estimated when five or fewer patients were exposed. Matching, data imputation, and statistical analyses were performed using IBM SPSS statistics version 27.0.

## 3. Results

**Figure 2:**
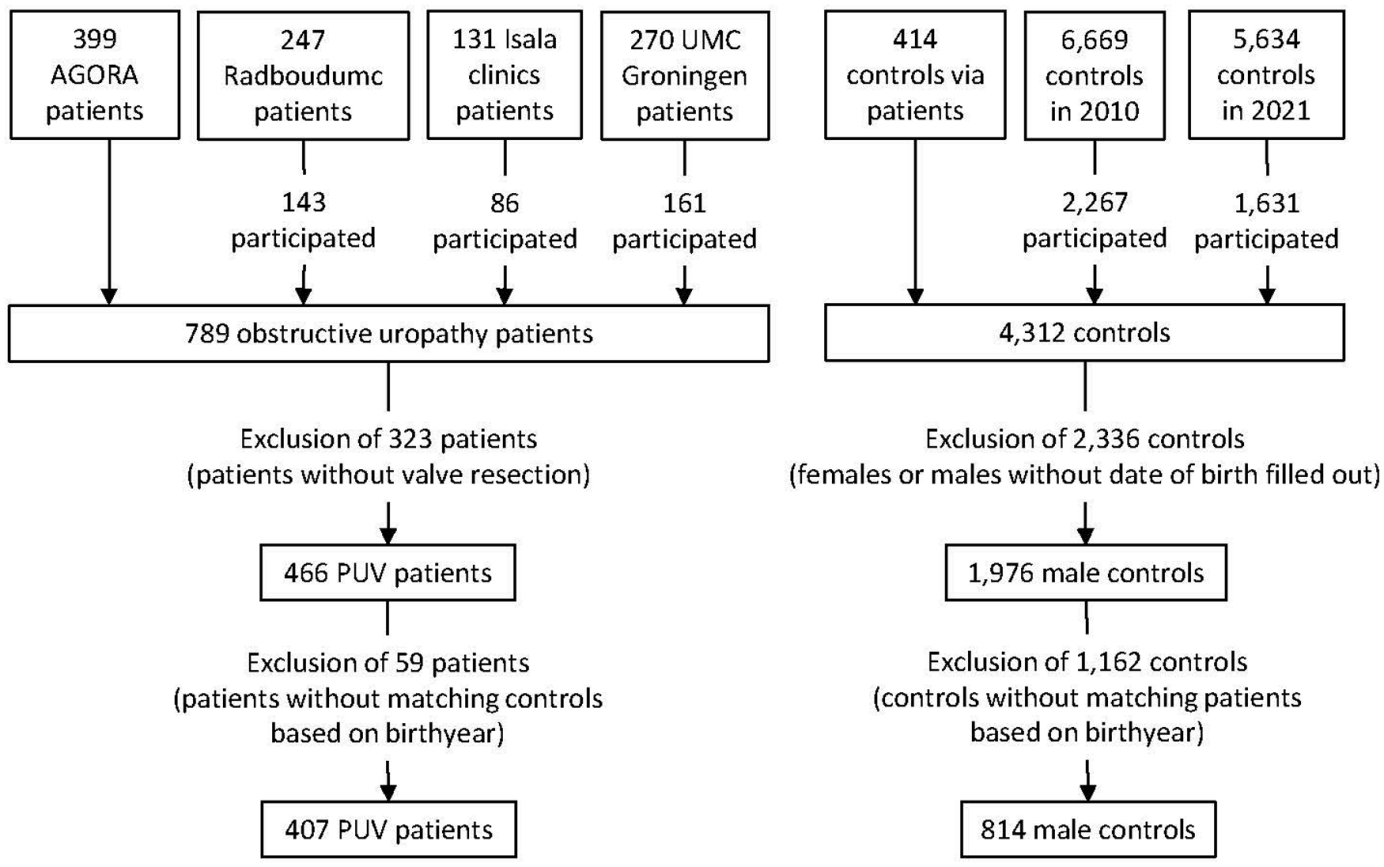
Flowchart showing the selection and in- and exclusion of patients and controls in the current study. PUV, posterior urethral valves.

Figure 2 shows the inclusion and selection of patients and controls. For our studies on obstructive uropathy, 399 eligible patients were included in the AGORA data- and biobank. We identified 247 additional obstructive uropathy patients in the Radboudumc, 131 in the Isala clinics, and 270 in the UMC Groningen. Of these, 143 (53%), 86 (66%) and 161 (60%) patients, respectively, responded positively and participated. We excluded 323 patients who did not have a valve resection, resulting in 466 PUV patients for the current study. A total number of 6,669 and 5,634 control families were asked to fill out the AGORA questionnaires in 2010 and 2021, respectively. Of these, 2,267 (34%) and 1,631 (29%) responded. In addition, the parents of 414 healthy children recruited by parents of patients completed the questionnaire online. We selected the 1,976 male controls without major birth defects for which at least the question about year of childbirth was filled out. Each patient was matched to two controls born in the same year to account for differences in year of childbirth between patients and controls. Therefore, we had to exclude all 35 patients born between 1981 and 1990 and 24 patients born in later years due to a lack of controls from those birth years. As a result, we performed our analyses with 407 PUV patients and 814 male controls. Part of this study population (*N* = 127 patients) was used in a previous study in which we investigated 562 CAKUT patients, but also performed subgroup analyses for separate phenotypes including PUV (6). Table 1 shows that most patients and controls in the current study were born from 2000 onwards. Mothers of controls reported slightly more often to be highly educated and much more often completed the questionnaire on paper, with only 7% of control parents and 40% of patient parents completing the questionnaire online. Time between childbirth and completion of the questionnaires was similar for patients and controls, and generally 5 years or longer.

**Table 1:** Characteristics of patients with posterior urethral valves and healthy population-based controls matched on year of childbirth.

	Patients (*n* = 407)	Matched controls (*n* = 814)
Year of childbirth		
<2000	126 (31%)	252 (31%)
2000–2004	123 (30%)	246 (30%)
2005–2009	109 (27%)	218 (27%)
2010–2014	45 (11%)	90 (11%)
≥2015	4 (1%)	8 (1%)
Maternal ethnicity		
European	398 (98%)	782 (96%)
Other	8 (2%)	31 (4%)
Maternal education level		
Low	61 (15%)	124 (15%)
Intermediate	195 (48%)	337 (42%)
High	149 (37%)	349 (43%)
Completion of questionnaire		
On paper	243 (60%)	758 (93%)
Online	164 (40%)	56 (7%)
Time from birth to completion of questionnaire		
<2 years	32 (8%)	48 (6%)
2–4.99 years	44 (11%)	120 (15%)
≥5 years	331 (81%)	646 (79%)

The results of the univariable and multivariable conditional logistic regression analyses are displayed in Table 2. A positive family history of CAKUT and low maternal age (<25 years) were associated with PUV development in both univariable and multivariable analyses (adjusted odds ratios (aOR) 3.3 and 1.7 with 95% confidence intervals (95% CI) 1.4–7.7 and 1.0–2.8, respectively), whereas higher maternal age (≥35 years) was associated with a lower risk (aOR: 0.7 95% CI: 0.4–1.0). Maternal preexisting hypertension seemed to be associated with an increased PUV risk (aOR: 2.1 95% CI: 0.9–5.1), while maternal gestational hypertension seemed to be associated with a decreased risk (aOR: 0.6 95% CI: 0.3–1.0). Concerning use of ART, the aORs for the different techniques were all above 1, ranging from 1.4 to 2.0, but with very wide 95% CIs including one. Slightly increased aORs of 1.4 with 95% CIs including one were also observed for women with underweight, obesity, or gestational diabetes. None of the other factors studied were associated with PUV development.

**Table 2:** Crude and adjusted odds ratios for the associations between potential risk factors and posterior urethral valves.

	Patients (*n* = 407)	Controls (*n* = 814)	cOR^a^	aOR^b^	95%CI
Family history of CAKUT					
No	370 (95%)	722 (99%)	ref	ref	
Yes	18 (5%)	10 (1%)	3.7	3.3	1.4–7.7
Season of conception					
Winter	94 (24%)	200 (25%)	ref	ref	
Spring	107 (28%)	210 (26%)	1.1	1.0	0.7–1.5
Summer	90 (23%)	163 (20%)	1.1	1.1	0.7–1.7
Fall	99 (25%)	229 (29%)	0.9	0.8	0.5–1.2
Gravidity					
First pregnancy	181 (45%)	320 (40%)	ref	ref	
Subsequent pregnancy	219 (55%)	483 (60%)	0.8	1.0	0.8–1.4
Subfertility					
Fertile	312 (81%)	607 (82%)	ref	ref	
Subfertile without ART	45 (12%)	103 (14%)	0.9	0.9	0.6–1.4
IUI without hormones	7 (2%)	7 (1%)	2.3	2.0	0.6–7.3
Hormones with or without IUI	14 (3%)	15 (2%)	1.8	1.7	0.7–3.9
IVF/ICSI	8 (2%)	11 (1%)	1.6	1.4	0.5–3.6
Maternal age					
<25 year	44 (11%)	51 (6%)	1.9	1.7	1.0–2.8
25–34 year	296 (75%)	594 (74%)	ref	ref	
≥35 year	57 (14%)	162 (20%)	0.7	0.7	0.4–1.0
Maternal BMI					
Underweight (<18.5 kg/m^2^)	16 (4%)	25 (3%)	1.3	1.4	0.7–3.1
Normal (18.5–24.9 kg/m^2^)	257 (68%)	549 (72%)	ref	ref	
Overweight (25–29.9 kg/m^2^)	76 (20%)	141 (18%)	1.1	1.2	0.8–1.7
Obese (≥30 kg/m^2^)	30 (8%)	51 (7%)	1.3	1.4	0.8–2.5
Maternal diabetes					
No diabetes	382 (97%)	787 (98%)	ref	ref	
Gestational diabetes	11 (3%)	15 (2%)	1.4	1.4	0.6–3.4
Preexisting diabetes	1 (0%)	1 (0%)	^	^	^
Hypertension					
No hypertension	347 (88%)	689 (87%)	ref	ref	
Gestational hypertension	31 (8%)	88 (11%)	0.7	0.6	0.3–1.0
Preexisting hypertension	15 (4%)	16 (2%)	2.0	2.1	0.9–5.1
Smoking					
No smoking	307 (77%)	654 (80%)	ref	ref	
Smoking in etiological period^c^	61 (15%)	104 (13%)	1.3	1.2	0.8–1.9
Smoking prior to this period^d^	31 (8%)	54 (7%)	1.3	1.1	0.6–2.0
Alcohol					
No alcohol	252 (63%)	464 (57%)	ref	ref	
Alcohol in etiological period^c^	31 (8%)	77 (10%)	0.8	1.0	0.6–1.7
Alcohol prior to this period^d^	117 (29%)	270 (33%)	0.8	1.0	0.7–1.3
Folic acid supplementation					
No supplementation	108 (29%)	218 (28%)	ref	ref	
Use as recommended^e^	155 (42%)	321 (42%)	1.1	1.3	0.8–1.9
Suboptimal use^f^	108 (29%)	230 (30%)	1.0	0.9	0.6–1.4

cOR, crude odds ratio; aOR, adjusted odds ratio; CI, confidence interval; ref, reference; BMI, body mass index; ART, artificial reproductive technique; IUI, intrauterine insemination; IVF, *in vitro* fertilization; ICSI, intracytoplasmic sperm injection; CAKUT, congenital anomalies of the kidney and urinary tract.

For ORs printed in bold, the 95% CI did not include one before rounding.

^Not calculated since ≤five patients were exposed.

^a^ Unadjusted odds ratio calculated on original matched dataset with missing values.

^b^ Adjusted odds ratio, adjusted for minimal set of confounders determined using directed acyclic graph (DAG) plus a variable indicating whether the questionnaire was completed online or on paper, and calculated using the 10 imputed datasets.

^c^ Use in the etiological period was defined as using before pregnancy and not stopping before the sixth week of pregnancy.

^d^ Use prior to the etiological period was defined as any use in the three months before or during the first five weeks of pregnancy only.

^e^ Use as recommended is initiation before pregnancy and not stopping before the 8th week of pregnancy.

^f^ Suboptimal use was defined as usage in the four weeks before pregnancy or in the first eight weeks of pregnancy, but not the entire period.

## 4. Discussion

In the current case-control study with 407 PUV patients and 814 population based-controls, we identified family history of CAKUT and low maternal age as potential risk factors for PUV development, whereas higher maternal age was associated with a lower risk. Maternal preexisting hypertension seemed to increase PUV risk, while gestational hypertension seemed to decrease this risk. Concerning use of ART, the aORs for the different techniques were all above one, but with very wide 95% CIs including one.

This retrospective study included patients and controls born from 1990 onwards. Because several exposures, such as use of folic acid supplements and smoking during pregnancy, changed considerably in the past three decades, we used matching on year of childbirth to prevent confounding by changes over time. A strength of our study is the large number of patients with a well-defined phenotype, in contrast to previous studies that often included a broad range of CAKUT patients. This is especially important as it has been debated whether PUV is part of the CAKUT phenotype spectrum and the possibility of a different underlying cause has been suggested for this specific phenotype (20). Another strength is the availability of information on many potential risk factors and confounders. We used DAGs to select minimally needed sets of confounders for each of the prespecified risk factors, which increases statistical efficiency and avoids overcorrection introducing bias (21). By displaying all possible relations in the DAG, we made the assumptions about causal associations between variables explicit. Despite these strengths, causality cannot be proven with a retrospective observational study like ours.

The time between childbirth and completion of the questionnaire was 5 years or more for ∼80% of the population. Although the time to completion was comparable among mothers of patients and controls, the large time gap may have resulted in some recall errors. The majority of factors that we studied, however, represent maternal or pregnancy characteristics that can easily be retrieved (season of conception, gravidity, subfertility, maternal age, and parental CAKUT) or were clear health conditions (diabetes and hypertension). Therefore, we believe that recall errors for these factors would have been limited and most likely non-differential, only leading to slight underestimation of the odds ratios. The lifestyle habits that we investigated (maternal BMI, smoking, alcohol, and folic acid use) are usually easy to remember in the well-defined period of pregnancy as well, but some differential recall bias cannot be ruled out for these factors. In addition, the way the questionnaire was completed (online or on paper) may have resulted in differential responses. However, the questions were asked exactly the same in both versions and we corrected all of our analyses for the variable indicating online or on paper completion.

PUV is considered to be a multifactorial disorder involving both non-genetic and genetic factors, as familial forms (22) as well as affected sib-pairs have been described (23) and a classical twin study found higher concordance rates among monozygotic compared to dizygotic twin pairs (5). The fact that a family history of CAKUT was the strongest risk factor found in our study supports this hypothesis. Genetic studies previously showed that copy number variants (CNVs) could play a role (24, 25), while associations with common variants have also been reported (26, 27).

We observed an association between PUV and younger maternal age and a protective effect of more advanced maternal age, whereas a previous study that focussed on patients with ureter, bladder, and urethra abnormalities found advanced maternal age to be a risk factor (7). Although the common thought is that advanced maternal age negatively impacts pregnancy outcomes (28), another study showed associations between maternal age and renal agenesis similar to our estimates (29). Therefore, further research into the role of maternal age is warranted.

Our study also points towards an association between PUV and maternal preexisting hypertension, while gestational hypertension seemed to decrease the risk. Gestational hypertension was defined as hypertension detected in the 20th week of pregnancy or later, well after the etiologically relevant period. Therefore, it seems unlikely that gestational hypertension reduces the risk of PUV, although an underlying condition predisposing to gestational hypertension may perhaps play a role. Preexisting hypertension was not associated with ureter, bladder and urethra abnormalities or with other CAKUT phenotypes in most previous studies (7, 30, 31), although the most recent study did find an increased risk (32). Maternal hypertension was consistently associated with hypospadias (33), however, which also is a defect of the urethra and may partly share its' aetiology with PUV. For hypospadias, the association with maternal hypertension was hypothesized to be caused by placental dysfunction in early pregnancy (33). Whether a similar mechanism may be involved in PUV, warrants further research.

Maternal subfertility was not associated with PUV in our study, whereas use of ART may be although the confidence intervals were very wide and included one. An association with ART would be consistent with the results of our previous study (6) and with the slightly elevated risk of urogenital anomalies after ART reported by an Australian registry study (34). ART may lead to congenital defects via the hormonal treatment interfering with the foetal endocrine system, via the *in vitro* procedures of IVF/ICSI involving a risk of epigenetic defects (35), or via factors underlying the subfertility and the need to use ART.

Weak potential associations were also observed for obesity and gestational diabetes, but again the wide 95% CIs included one. Most previous studies on CAKUT in general but including patients with obstructive uropathy or urethral malformations showed associations with maternal obesity (6, 8, 9) and gestational diabetes (6–8, 10). One study did not find an association with obesity (11) and in two studies the association with obesity disappeared when focussing on patients with lower urinary tract anomalies or PUV only (6, 9). Regarding gestational diabetes, Shnorhavorian et al*.* found a stronger effect in the phenotypes affecting the kidneys (7), whereas we previously showed that this effect was stronger in PUV patients (6).

We did not find any association with season of conception, smoking, or alcohol use, which is consistent with most previous studies (6, 7, 12). We were unable to confirm the previously reported association with gravidity (7, 32) and the protective effect of the use of folic acid supplements or folic-acid containing multivitamins (6, 13, 14). The latter is consistent with our previous finding of the protective effect of folic acid among CAKUT patients disappearing when focussing on PUV patients only (6).

In conclusion, our study shows that family history of CAKUT and low maternal age are associated with an increased risk of PUV development, while higher maternal age was associated with a lower risk. Maternal preexisting hypertension seemed to be associated with an increased PUV risk as well. Both preexisting and gestational hypertension and maternal age as well as the possible role of ART require further research. We suggest that future studies on the etiology of CAKUT should stratify on different phenotypes.

## Data Availability

The raw data supporting the conclusions of this article will be made available by the authors, without undue reservation.
